# Gliosarcoma: A Multi‐Institutional Analysis on Clinical Outcomes and Prognostic Factors

**DOI:** 10.1002/cam4.70347

**Published:** 2024-11-15

**Authors:** Siyer Roohani, Maximilian Mirwald, Felix Ehret, Christoph Fink, Laila König, Jana Käthe Striefler, Noelle Samira Jacob, Ilinca Popp, Johannes Steffel, Jolina Handtke, Noa Marie Claßen, Titus Rotermund, Daniel Zips, Peter Vajkoczy, Ulrich Schüller, Mateusz Jacek Spałek, David Kaul

**Affiliations:** ^1^ Department of Radiation Oncology Charité—Universitätsmedizin Berlin, Corporate Member of Freie Universität Berlin and Humboldt‐Universität zu Berlin Berlin Germany; ^2^ BIH Charité Junior Clinician Scientist Program Berlin Institute of Health at Charité—Universitätsmedizin Berlin, BIH Biomedical Innovation Academy Berlin Germany; ^3^ German Cancer Consortium (DKTK) partner site Berlin, a partnership between DKFZ and Charité‐Universitätsmedizin Berlin, Germany Heidelberg Germany; ^4^ Department of Radiation Oncology University Hospital Heidelberg Heidelberg Germany; ^5^ National Center of Radiation Oncology Heidelberg Institute of Radiation Oncology (HIRO) Heidelberg Germany; ^6^ National Center for Tumor Diseases (NCT) Heidelberg Germany; ^7^ Department of Internal Medicine II, Oncology/Hematology/BMT/Pneumology University Medical Center Hamburg‐Eppendorf Hamburg Germany; ^8^ Department of Radiation Oncology Medical Center University of Freiburg, Faculty of Medicine Freiburg Germany; ^9^ German Cancer Consortium (DKTK) Partner Site Freiburg German Cancer Research Center (DKFZ), Im Neuenheimer Feld Heidelberg Germany; ^10^ Department of Neurosurgery Charité—Universitätsmedizin Berlin, Corporate Member of Freie Universität Berlin and Humboldt‐Universität zu Berlin Berlin Germany; ^11^ Department of Pediatric Hematology and Oncology University Medical Center Hamburg‐Eppendorf Hamburg Germany; ^12^ Research Institute Children's Cancer Center Hamburg Hamburg Germany; ^13^ Institute of Neuropathology, University Medical Center Hamburg‐Eppendorf Hamburg Germany; ^14^ Department of Soft Tissue/Bone Sarcoma and Melanoma Maria Sklodowska‐Curie National Research Institute of Oncology Warsaw Poland; ^15^ Department of Radiotherapy I Maria Sklodowska‐Curie National Research Institute of Oncology Warsaw Poland

**Keywords:** gliosarcoma, *MGMT*, outcomes, prognostic factors, radiotherapy, surgery, *TP53*

## Abstract

**Purpose:**

This study describes oncological outcomes and investigates prognostic factors for patients with gliosarcomas (GSM).

**Methods:**

Histopathologically confirmed GSM patients who underwent treatment at five European institutions were retrospectively analyzed.

**Results:**

We analyzed 170 patients with a median clinical follow‐up time of 9.2 months. The majority received surgery (94.1%), postoperative radiotherapy (pRT, 81.8%), and temozolomide (TMZ)‐based postoperative chemotherapy (66.5%). The median overall survival (OS) and progression‐free survival (PFS) were 12.3 and 6.6 months, respectively. In the multivariable Cox regression analysis (MVA), the following factors were significantly associated with OS: age per year (hazard ratio (HR): 1.03, *p* < 0.001), subtotal resection (STR) versus biopsy only (HR: 0.15, *p* = 0.018), gross total resection (GTR) versus biopsy only (HR: 0.13, *p* = 0.011), pRT versus no pRT (HR: 0.20, *p* < 0.001), postoperative TMZ‐based chemotherapy versus no postoperative chemotherapy (HR: 0.44, *p* = 0.003), *MGMT* promoter non‐methylated versus methylated (HR: 1.79, *p* = 0.05), and tumor diameter per cm (HR: 1.15, *p* = 0.046). For PFS, the following factors were significantly associated in the MVA: GTR versus biopsy only (HR: 0.19, *p* = 0.026), pRT versus no pRT (HR: 0.36, *p* = 0.006), postoperative TMZ‐based chemotherapy vs. no postoperative chemotherapy (HR: 0.39, *p* < 0.001), *MGMT* promoter status unknown versus methylated (HR: 1.69, *p* = 0.034), and tumor diameter per cm (HR: 1.18, *p* = 0.016). Sex, primary or secondary GSM, and *TP53* mutational status were not significantly associated with OS or PFS.

**Conclusions:**

Trimodal therapy comprising surgical resection, pRT and TMZ‐based chemotherapy appears to have the most beneficial effect on survival in GSM patients. Smaller tumor size, younger age and methylated *MGMT* promoters are associated with improved survival. To our knowledge, this is the largest multi‐institutional cohort study investigating outcomes and prognostic factors for GSM.

**Abbreviations**
EQD2: equivalent dose in 2 Gy fractions
GSM: gliosarcoma
GTR: gross total resection
HR: hazard ratio
IDH: Isocitrate dehydrogenase
IQR: interquartile range
*MGMT*
: O6‐methylguanine‐DNA methyltransferase
MRI: magnetic resonance imaging
N/A: not available
OS: overall survival
PFS: progression‐free survival
pRT: postoperative radiotherapy
RT: radiotherapy
SEER: surveillance, epidemiology, and end results
STR: subtotal resection
TMZ: temozolomide
TTF: Tumor‐treating fields

## 1 Introduction

Gliosarcomas (GSM) are rare, malignant primary central nervous system tumors and distinct subtypes of glioblastoma histologically characterized by glial and sarcomatous components [1, 2]. GSM,ee which account for 2%–5% of glioblastomas, are typically located in the temporal lobe of patients in their late 50s to early 60s and have a male predominance [1, 3, 4, 5]. Primary GSM arising de‐novo are distinguished from secondary GSM, which develop after transformation from previous glioblastoma, anaplastic glioma, or low‐grade glioma [2, 5, 6, 7]. Although strong supporting evidence is lacking, GSM are typically managed according to the trimodal treatment of glioblastoma comprising maximal safe resection, postoperative temozolomide (TMZ)‐based radiochemotherapy, and sequential TMZ chemotherapy [8, 9]. While some studies report even poorer prognoses for GSM than for glioblastoma, with median overall survival (OS) times of 6–15 months, others did not find significant outcome differences [4, 5, 10, 11, 12]. Secondary GSM, however, are uniformly reported as carrying distinct genetic and epigenetic profiles leading to unfavorable prognoses compared to primary GSM [7, 13, 14, 15]. Multiple case series and retrospective cohort studies of up to 94 patients have described TMZ chemotherapy, radiotherapy (RT), primary GSM (vs. secondary), and gross total resection (GTR) as positive prognostic factors for OS [11, 13, 16, 17]. This study aims to clinically characterize and identify prognostic factors for oncological outcomes in a multi‐institutional cohort of GSM patients published to date.

## 2 Methods

This retrospective multi‐institutional cohort study included patients with histopathologically confirmed diagnoses of GSM, who received treatment at five European institutions between 1984 and 2023. We reviewed data on the patient characteristics, imaging, pathology, surgical, oncological, and RT treatment characteristics and oncological outcome data. Endpoints included OS and progression‐free survival (PFS). OS was defined as the time from surgery to death by any cause. PFS was defined as histopathological or radiographic evidence of disease progression after surgery on follow‐up magnetic resonance imaging (MRI) assessed by a board‐certified radiologist or death by any cause. GTR was defined as a surgical resection without visual residual enhancing tumor in the postoperative MRI, while subtotal resection (STR) was defined as a surgical resection with visual residual enhancing tumor in the postoperative MRI. Clinical follow‐up was calculated from the date of surgery until the last clinical visit. Radiographic follow‐up was calculated from the date of surgery until the last available MRI. Patients were censored at the last available follow‐up if no disease progression or death was observed. Data on survival status were obtained from the tumor registries of the participating centers.

For descriptive statistics, ranges, means, medians, standard deviations, and interquartile ranges for continuous variables were used. OS and PFS were assessed using the Kaplan–Meier estimator. Multivariable Cox regression was performed to analyze the influence of various factors on OS and PFS. A *p* ≤ 0.05 was considered statistically significant. The proportional hazard assumption was tested with Schoenfeld residuals. Statistical analysis was performed and figures created with GraphPad Prism v.9.3.1 (GraphPad Software, San Diego, CA, USA) and STATA MP 16.0 (StataCorp, College Station, TX, USA). The study was approved by the institutional review board (EA1/072/23).

## 3 Results

### 3.1 Patient and Treatment Characteristics

Patient and treatment characteristics are depicted in Table 1. In total, 170 patients were analyzed. The majority of patients (98.2%) was treated between 2000 and 2023. The median age at primary diagnosis was 58 years (range: 8–86 years) with a slight male predominance (56.5%). The median tumor diameter at initial diagnosis was 4.6 cm. Out of 170 cases, 153 (90.0%) were primary GSM, while secondary GSM were found in 12 patients (7.1%) with previous glioblastoma, 4 patients (2.4%) with initial low‐grade glioma, and 1 patient (0.6%) with previous anaplastic glioma at the same tumor location. All tumors with available isocitrate dehydrogenase (IDH) status (60.6%) were wild‐type. The *TP53* gene was wild‐type in 4.7%, mutated in 32.4%, and not available in 62.9% of patients. The O6‐methylguanine‐DNA methyltransferase (*MGMT*) promoter status was methylated in 27.1%, non‐methylated in 19.4%, and not available in 53.5% of patients.

**TABLE 1:** Patient and treatment characteristics.

	All *N* = 170	
	*N*	%
Demographics		
Age at first diagnosis, years		
Mean	57.5	
Median	58.0	
IQR	15.0	
Range	8.0‐86.0	
Sex		
Male	96	56.5
Female	74	43.5
Tumor characteristics		
Maximum tumor diameter, cm		
Mean	4.6	
Median	4.6	
IQR	2.2	
Range	1.0‐8.0	
Pathological diagnosis		
Primary GSM	153	90.0
Secondary GSM after previous GBM at same location	12	7.1
Secondary GSM after previous low‐grade glioma at same location	4	2.4
Secondary GSM after previous anaplastic glioma at same location	1	0.6
Metastases		
Total	10	5.9
Intracranial sites other than primary tumor	5	2.9
Carcinomatous meningitis	4	2.4
Distant organs	1	0.6
Neuropathology		
IDH		
Wild‐type	103	60.6
Information not available	67	39.4
*TP‐53*		
Wild‐type	8	4.7
Mutated	55	32.4
Information not available	107	62.9
*MGMT* Promoter methylation		
Methylated	46	27.1
Non‐methylated	33	19.4
Information not available	91	53.5
Treatment characteristics		
Surgery		
Surgical resection	160	94.1
Information not available	10	5.9
Extent of resection	160	100.0
Gross total resection	93	54.7
Subtotal resection	55	32.4
Biopsy	6	3.5
Information not available	16	9.4
Radiotherapy		
Postoperative radiotherapy	139	81.8
Definitive radiotherapy	3	1.8
No radiotherapy	16	9.3
Information not available	12	7.1
Postoperative radiotherapy	139	100.0
Dose per fraction, Gy		
Mean	2.2	
Median	2.0	
IQR	0.0	
Range	1.6‐5.0	
Fractions per day		
1 fraction per day	121	87.1
2 fractions per day	7	5.0
Information not available	11	7.9
Total dose, Gy		
Mean	54.0	
Median	60.0	
IQR	8.0	
Range	5.6‐67.0	
Equivalent dose in 2 Gy fractions, Gy		
Mean	54.4	
Median	60.0	
IQR	7.5	
Range	6.0‐67.1	
Chemotherapy		
Temozolomide‐based chemotherapy	113	66.5
No chemotherapy	44	25.9
Information not available	13	7.6
Temozolomide‐based chemotherapy	113	100
Concurrent to postoperative radiotherapy	27	23.9
Sequentially after postoperative radiotherapy	7	6.2
Concurrent and sequentially after postoperative radiotherapy	79	69.9
Trimodal therapy (gross total or subtotal resection + postoperative radiotherapy + temozolomide chemotherapy)		
Yes	102	60
No	68	40

Abbreviations: GBM = glioblastoma, GSM = gliosarcoma, IQR = interquartile range, *MGMT* = O6‐methylguanine‐DNA methyltransferase, IDH = Isocitrate dehydrogenase.

In total, 160 patients (94.1%) received surgery as the initial therapy; of which 93 (54.7%) were GTR, 55 (32.4%) were STR, and 6 (3.5%) were biopsies. Postoperative radiotherapy (pRT) was utilized in 139 (81.8%) patients, while 16 (9.3%) did not receive radiotherapy. Three patients (1.8%) received definitive RT after biopsy. The median pRT dose per fraction was 2.0 Gy once daily to a median total dose of 60.0 Gy. TMZ‐based chemotherapy was administered to 113 (66.5%) patients, while 44 (25.9%) patients did not receive chemotherapy. Overall, 102 patients (60%) received a trimodal therapy comprising GTR or STR with pRT and TMZ‐based chemotherapy and 68 patients (40%) did not.

### 3.2 Oncological Outcomes

**FIGURE 1:**
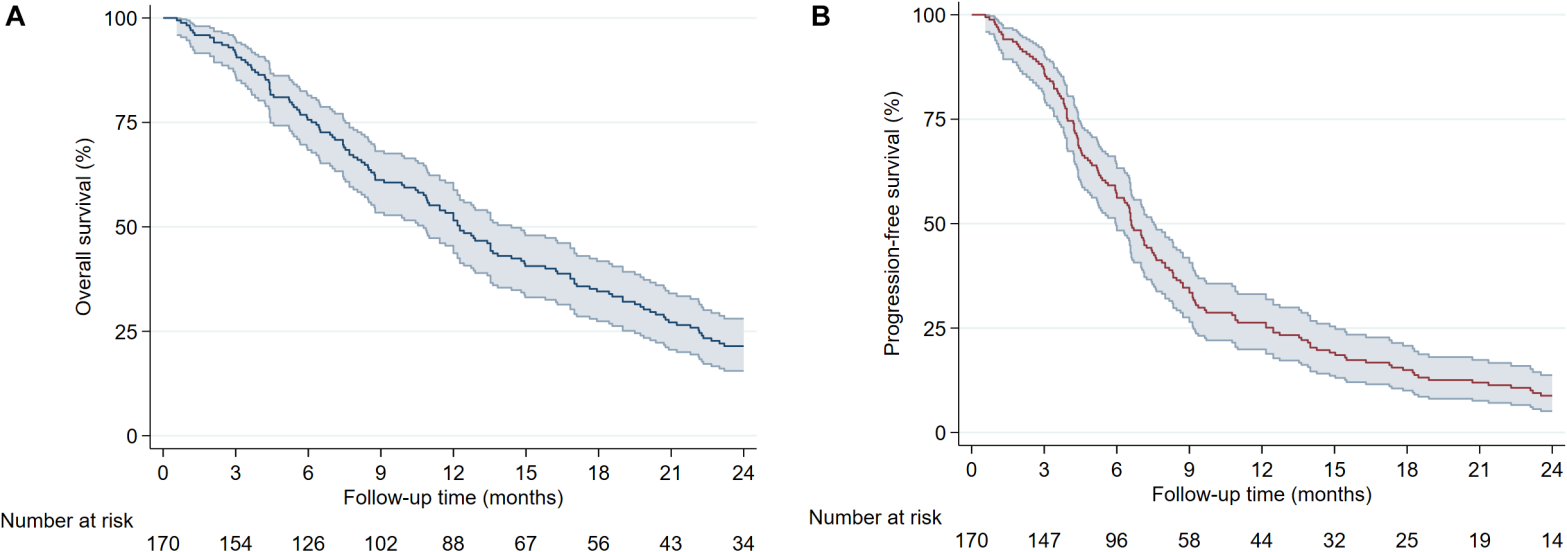
Overall survival (A) and progression‐free survival (B) in the entire study cohort.

Oncological outcomes are summarized in Table 2. The median clinical and radiographic follow‐up periods were 9.2 and 6.7 months, respectively. The median OS was 12.3 months (95% confidence interval: 10.8–14.4 months). The 3‐month, 6‐month, 1‐year, and 2‐year OS rates were 91.6%, 75.3%, 52.8%, and 20.7%, respectively (Figure 1A). The median PFS was 6.6 months (95% confidence interval: 6.0–7.5 months). The 3‐month, 6‐month, 1‐year, and 2‐year PFS rates were 87.0%, 57.4%, 26.3%, and 8.8%, respectively (Figure 1B).

**TABLE 2:** Oncological outcomes.

	Median	Mean	IQR	Range
Clinical follow‐up (months)	9.2	15.5	15.6	0.1–159.6

	Median (months)	3 months (%)	6 months (%)	1 year (%)	2 years (%)
Overall survival	12.3 (CI: 10.8–14.4)	91.6	75.3	52.8	20.7
Progression‐free survival	6.6 (CI: 6.0–7.5)	87.0	57.4	26.3	8.8

Abbreviations: CI = 95% confidence interval, IQR = interquartile range.

### 3.3 Multivariable Cox Regression Analysis

**FIGURE 2:**
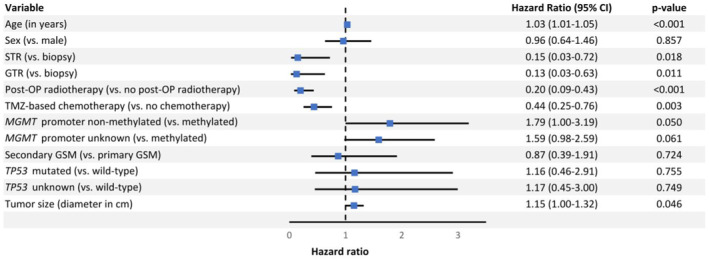
Multivariable Cox regression analysis for overall survival. CI = Confidence interval; GSM = Gliosarcoma; GTR = Gross total resection; MGMT = O6‐methylguanine‐DNA methyltransferase; STR = Subtotal resection, TMZ = Temozolomide.

In the multivariable Cox regression analysis, the following factors were significantly associated with OS (Figure 2): age per year (hazard ratio (HR): 1.03, *p* < 0.001), STR versus biopsy only (HR: 0.15, *p* = 0.018), GTR versus biopsy only (HR: 0.13, *p* = 0.011), pRT versus no pRT (HR: 0.20, *p* < 0.001), postoperative TMZ‐based chemotherapy versus no postoperative chemotherapy (HR: 0.44, *p* = 0.003), *MGMT* promoter non‐methylated versus methylated (HR: 1.79, *p* = 0.05), and tumor diameter per cm (HR: 1.15, *p* = 0.046). Formally *MGMT* promoter status unknown versus methylated (HR:1.59, *p* = 0.061) was not significantly associated with OS. Sex, primary or secondary GSM, and *TP53* mutational status were also not significantly associated with OS.

**FIGURE 3:**
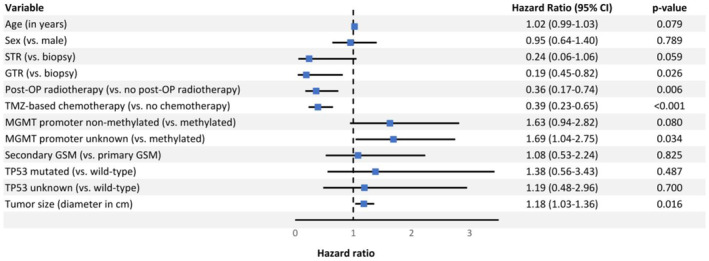
Multivariable Cox regression analysis for progression‐free survival. CI = Confidence interval; GSM = Gliosarcoma; GTR = Gross total resection; MGMT = O6‐methylguanine‐DNA methyltransferase; STR = Subtotal resection, TMZ = Temozolomide.

For PFS, the following factors were significantly associated in the multivariable Cox regression analysis (Figure 3): GTR versus biopsy only (HR: 0.19, *p* = 0.026), pRT versus no pRT (HR: 0.36, *p* = 0.006), postoperative TMZ‐based chemotherapy versus no postoperative chemotherapy (HR: 0.39, *p* < 0.001), *MGMT* promoter status unknown versus methylated (HR: 1.69, *p* = 0.034), and tumor diameter per cm (HR: 1.18, *p* = 0.016). Formally, age (HR: 1.02, *p* = 0.079), STR versus biopsy only (HR: 0.24, *p* = 0.059), and *MGMT* promoter non‐methylated versus methylated (HR: 1.63, *p* = 0.08) were not significantly associated with PFS. Sex, primary or secondary GSM, and *TP53* mutational status were also not significantly associated with PFS.

### 3.4 Subgroup Analyses

**FIGURE 4:**
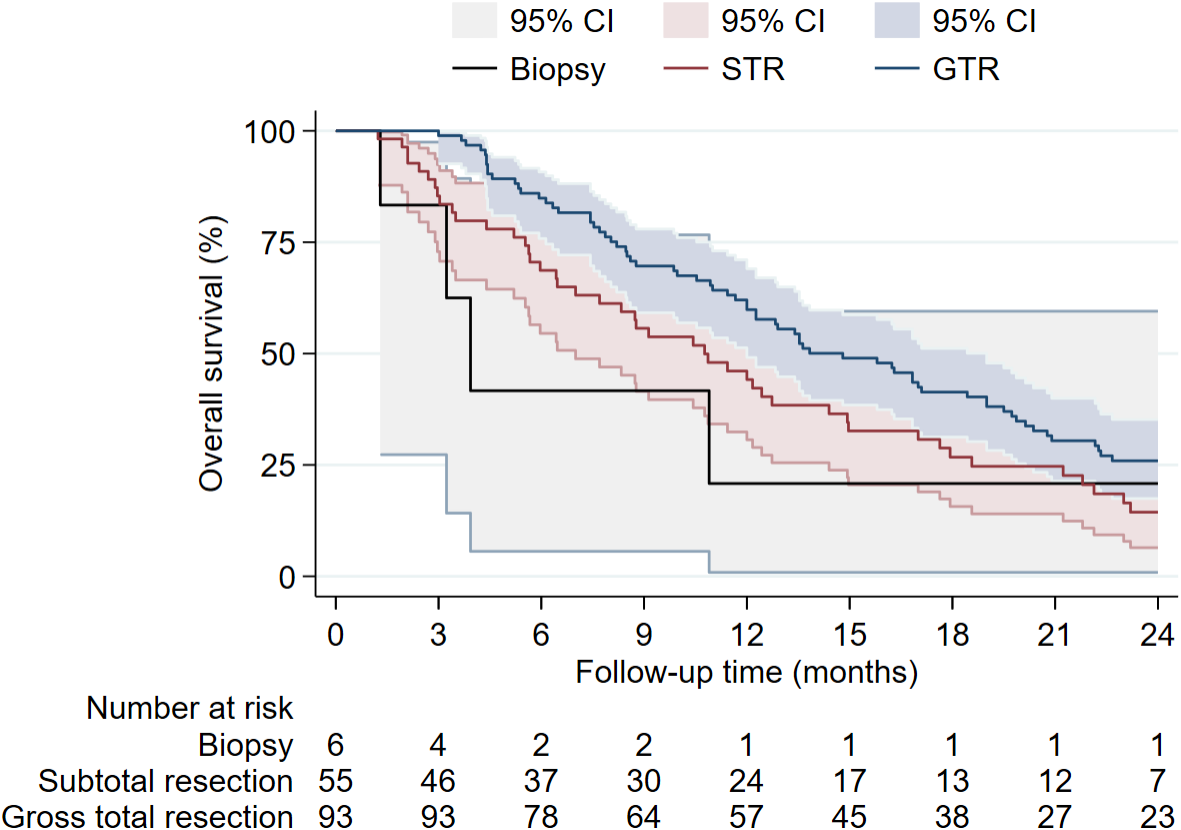
Overall survival in patients after subtotal resection, gross total resection and biopsy only.

The Kaplan–Meier estimate stratified by extent of resection is displayed in Figure 4. Patients who had a biopsy only had a shorter median OS of 3.9 months compared to 10.8 months after STR, and 14.8 months in patients after GTR.

**FIGURE 5:**
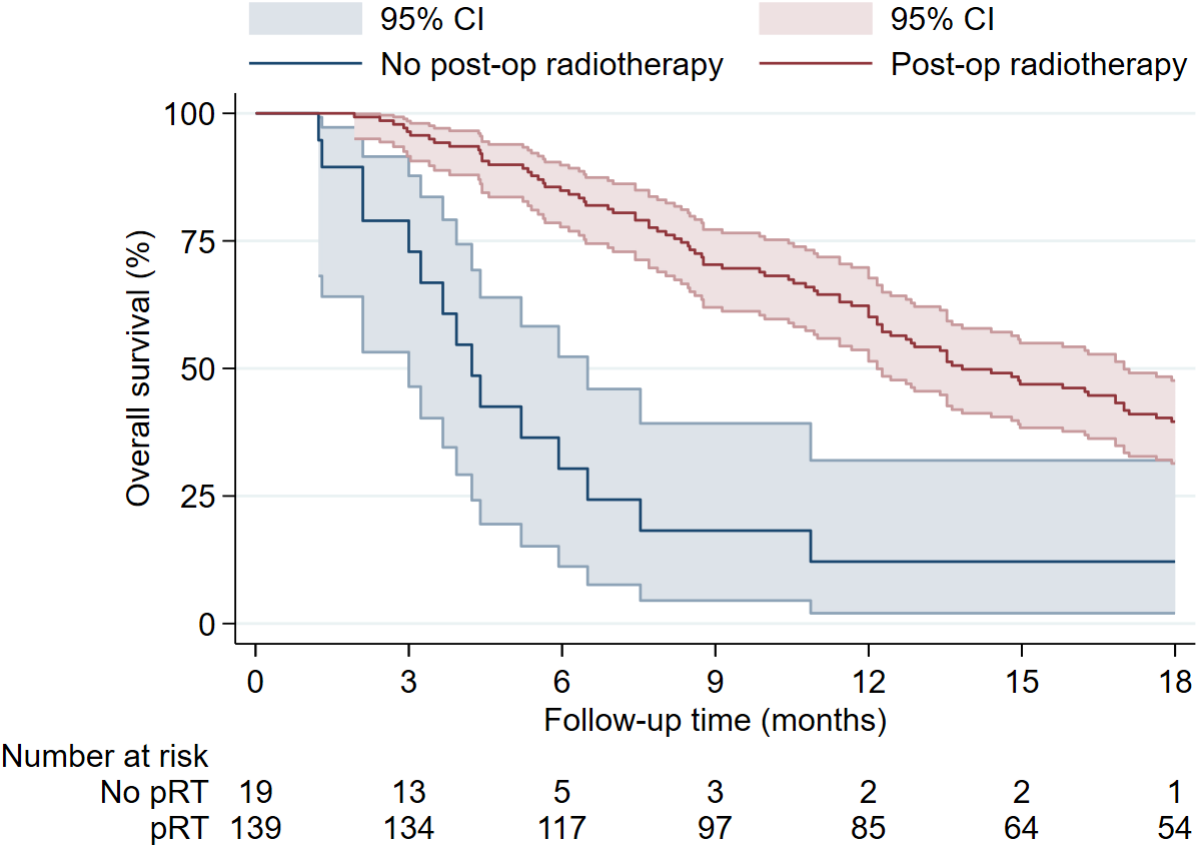
Overall survival in patients with and without postoperative radiotherapy.

**FIGURE 6:**
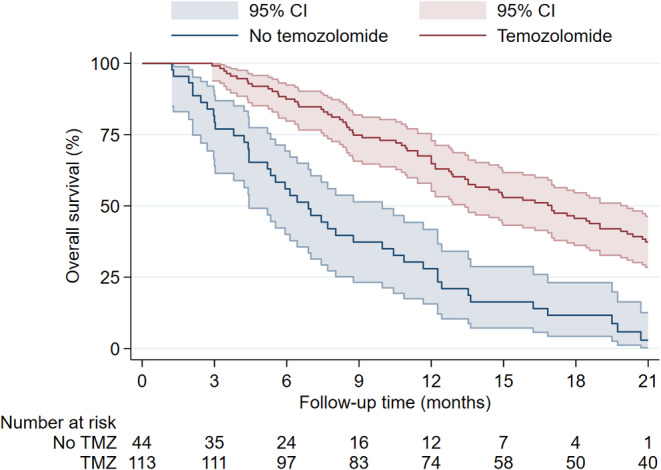
Overall survival in patients with and without postoperative temozolomide‐based chemotherapy.

**FIGURE 7:**
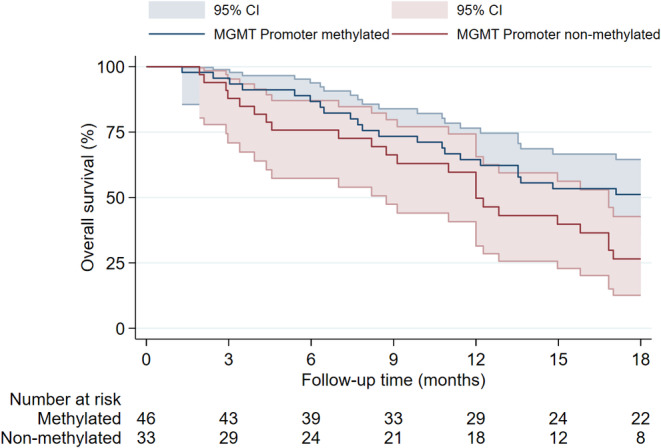
Overall survival in patients with *MGMT* promoter methylated and non‐methylated gliosarcomas.

**FIGURE 8:**
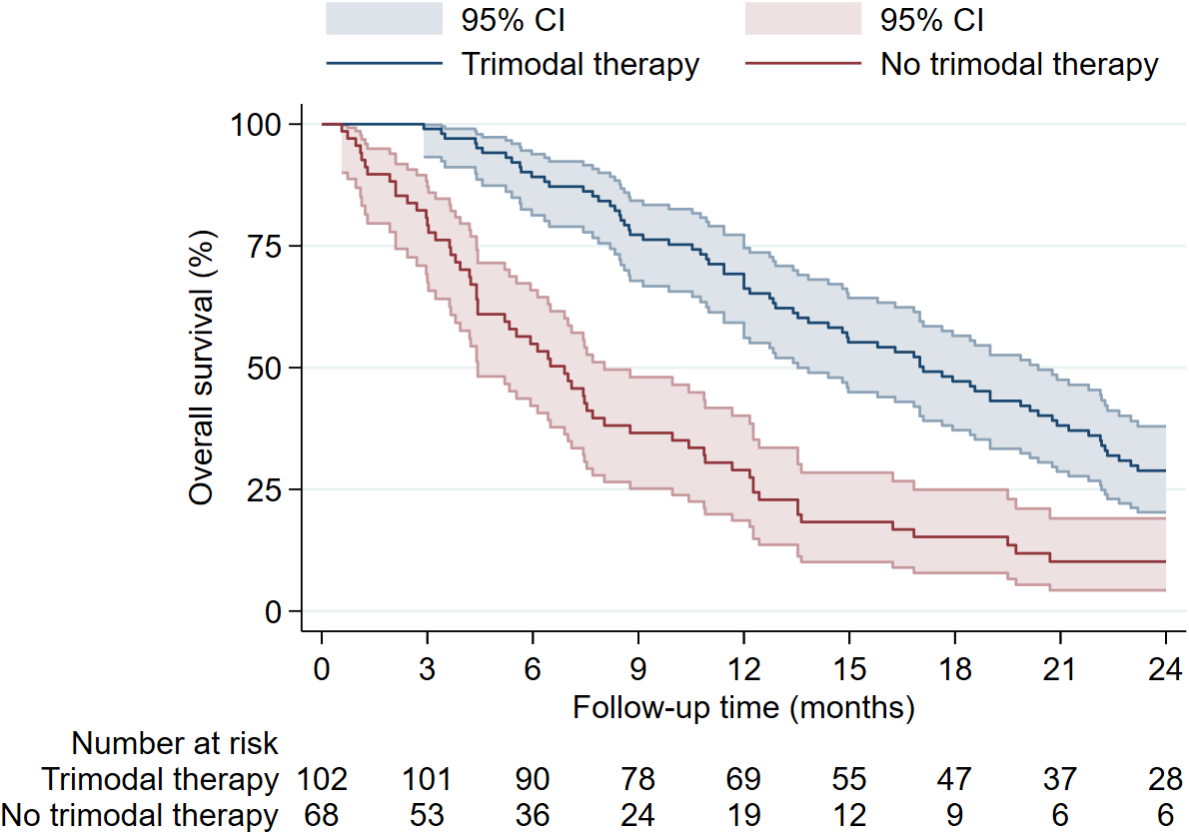
Overall survival in patients treated with and without trimodal therapy.

Figure 5 shows the Kaplan–Meier estimate of patients with and without pRT. Patients who received pRT showed a longer median OS of 13.8 months compared to 4.2 months in patients without pRT. Similarly, patients with postoperative TMZ‐based chemotherapy showed an increased OS compared to patients without postoperative TMZ‐based chemotherapy (Figure 6). The median OS was 17.0 months with postoperative TMZ‐based chemotherapy and 6.9 months without it. When stratified by *MGMT* promoter status, the survival curves showed diverging trends in favor of *MGMT* promoter methylation (Figure 7). The *MGMT* promoter methylated group had a median OS of 18.4 months, while the unmethylated group had 12.0 months. While primary and secondary GSM did show notable median OS differences of 12.7 months in primary and 7.5 months in secondary GSM, the factor was not significantly associated with OS or PFS in the multivariable analysis (Figures 2 and 3). Survival rates did not differ substantially between male and female patients (median OS 12.8 vs. 10.9 months, respectively) or *TP53* wild‐type and mutated GSMs (median OS of 10.9 vs. 13.5 months, respectively). Patients who underwent trimodal therapy showed a striking difference in OS to patients who did not. Median OS in the trimodal therapy group was 17.1 months and 6.9 months in the no trimodal therapy group (Figure 8).

## 4 Discussion

To our knowledge, this is the largest multi‐institutional analysis of GSM patients, comprising 170 patients from five European institutions. The prognosis of GSM with a median OS of 12.3 and median PFS of 6.6 months is poor. Although no prospective data or recommendations exist for the management of GSM, the aggressive trimodal therapy established for glioblastoma is associated with improved survival. Importantly, any surgical intervention, GTR or STR, was associated with improved survival over biopsy alone. Another similarity to glioblastoma is the prognostic value of the *MGMT* promoter status for survival. Tumor size and age are further important risk factors. The patient's sex, *TP53* mutational status, and the initial presentation with primary or secondary GSM were not associated with oncological outcomes.

Demographically, our findings confirm others in the literature. The patients' median age at diagnosis is typically in the late 50s and early 60s and has a slightly larger proportion of male than female patients [3, 11, 13, 17, 18, 19, 20, 21, 22]. Also, the oncological outcomes (median OS 12.3 months, median PFS 6.6 months) were in between previous studies, ranging from 9 to 17 months for OS and 5 to 8 months for PFS [3, 11, 13, 16, 17, 18, 23]. Moreover, the median tumor size at diagnosis of 4.6 cm in the present study was similarly found in other analyses [3, 18, 22].

### 4.1 Therapy Modalities

The beneficial survival effect of trimodal therapy detected herein confirms previous findings [21].

Moreover, any surgical intervention, GTR or STR, appears to be beneficial for OS over biopsy alone. At the same time, no significant outcome benefits of GTR over STR were evident. Consistent with our findings, other studies also underline the importance of GTR for survival; however, no study indicated an outcome benefit of GTR over STR [3, 11, 21, 24].

The present study further suggests a positive impact of pRT on OS and PFS, mostly utilizing a total median equivalent dose in 2 Gy fractions (EQD2) of 60 Gy. Castelli et al. also concluded that RT improves OS in 75 GSM patients receiving a total EQD2 of > 54 Gy [17]. The National Cancer Institute database analysis by Frandsen et al. and a study on 32 GSM patients by Perry et al. described a longer OS by pRT compared to non‐irradiated patients, although no results on multivariable analysis for pRT vs. no pRT were available [4, 21]. Similarly, in a Surveillance, Epidemiology, and End Results (SEER) database analysis by Kozak et al, pRT was a significant predictor for OS [3]. While database analyses carry significant limitations in terms of patient and treatment‐specific data, the studies do strengthen the hypothesis towards a benefit of pRT for GSM. pRT was also significantly associated with PFS in the present study, a finding that was not yet described in the literature. This may be due to the smaller sample sizes in other cohort studies and the limited follow‐up imaging data in larger database analyses. The positive impact of TMZ‐based postoperative chemotherapy on OS and PFS in our study was almost uniformly found in previous GSM studies, which underlines the similar clinical behavior to glioblastoma, where the addition of TMZ to pRT is standard of care and improves survival [8, 9, 11, 16, 17, 24].

Notably, the PFS curve in the present study did not plateau at any time point or with any therapy modality (Figure 1B). This finding is consistent with the literature and shows that GSM is currently an incurable disease which will eventually recur regardless of the aggressiveness of surgical intervention or postoperative radiochemotherapy [11, 16, 17, 24]. Still, the data suggest that the trimodal therapy may extend the survival time and should, therefore, be leveraged, whenever possible.

### 4.2 Prognostic Factors

Risk factors for survival identified herein were age, tumor size, and lack of *MGMT* promoter methylation. Age as a risk factor for survival in the present study was clearly supported by previous studies [3, 21, 22]. Data on tumor size was limited to one study by Pierscianek et al. on 56 GSM patients where tumor size was independently associated with worse OS [22]. In the SEER database analysis by Kozak et al. the tumor size was not a risk factor for OS; however, the informative value is limited as the tumor size in a large proportion of patients was unknown, as indicated by the authors [3]. One National Cancer Database analysis by Frandsen et al. found an improved survival associated with female sex, a finding which was not confirmed in our or other previous studies [21].

In 79 patients with available *MGMT* promoter status in our study, 46 (58.2%) were methylated and 33 (41.8%) non‐methylated and the latter had a significant negative impact on OS and almost reached significance for PFS. Moreover, in 91 patients with unknown *MGMT* promoter status, the hazard ratio for death was significantly increased, suggesting that the *MGMT* promoter status plays an important role for survival not only in glioblastoma but also in GSM. Previous literature did not find statistically significant differences between patients with methylated vs. non‐methylated *MGMT* promoter [11, 16, 21, 23, 25]. However, all studies were limited to small numbers of cases with available *MGMT* promoter status ranging between 11 and 47 patients. Moreover, most studies did show diverging trends in survival curves between both groups that may have reached statistical significance with increased patient numbers [11, 21, 25, 26]. While none of the aforementioned risk factors is influenceable, they should still be taken into account during the interdisciplinary therapy decision making process.

Although previous studies described secondary GSM to have worse oncological outcomes than primary GSM, this factor did not show a significant association in the multivariable analysis for OS or PFS herein [2, 6, 13, 14]. This finding is rather counterintuitive as secondary GSM are a selected patient cohort of heavily pretreated gliomas and, therefore, different from patients presenting with an initial diagnosis. However, no firm conclusions can be drawn from the small sample size of 17 secondary GSMs herein. Data on the underlying molecular profile of secondary GSM compared to primary GSM is limited to case reports and small case studies without striking differences between both entities [2, 27].

TP53 is one of the most important tumor suppressor proteins coded by the *TP53* gene and frequently mutated in a multitude of malignancies [28]. In the present study, 55 out of 63 tumors with available *TP53* mutation status (87.3%) carried a *TP53* mutation, which did not appear to have an impact on survival. Three studies also found similar *TP53* mutation rates (60.0%–73.4%) [13, 29, 30]. Only one analysis by Cho et al. suggested a survival benefit for wild‐type *TP53* GSM; however, the data is limited by the small sample size of 28 cases [29]. In other studies, the frequency of *TP53* mutations was lower and ranged between 24%–26%, similar to the *TP53* mutation rate found in glioblastoma [31, 32, 33, 34, 35]. No other studies observed an association with survival by *TP53* mutational status in GSM or glioblastoma [13, 36, 37]. *TP53* is not a mutation targetable with FDA‐approved drugs according to the OncoKB precision oncology database classification system [28, 38]. Still, GSM were more recently identified to carry gene mutations in five genes (*BRAF*, *EGFR*, *CDKN2A*, *NF1*, *and PTEN*) with high frequencies of 10%–50%, all of which are potentially amenable to targeted therapies [30, 38].

Compared to glioblastoma, our and previous data point out similarities in clinical behavior. Both are primary brain tumors and currently predominantly non‐curative disease entities where intensive trimodal therapy is beneficial for survival, but still with a similar limited prognosis [5, 8, 9, 21]. For GSM, our data shows that *MGMT* promoter methylation is also prognostically relevant and TMZ‐based chemotherapy improves survival, as it does for glioblastoma [8, 9]. Interestingly, however, GSM carry a distinct genomic profile compared to glioblastoma and soft tissue sarcomas as well [30]. This indicates the need for re‐classification of GSM as a separate disease entity. Moreover, the methylome‐based brain tumor classification may also aid in outlining GSM as a separate entity. So far, no data has been published on this topic since the introduction of methylome‐based brain tumor classification and the subsequent reclassification of brain tumors [1, 42]. To further investigate this, multi‐institutional collaborations are needed to decipher the molecular profile of GSM which can be the foundation for the research of GSM‐directed therapies.

Moreover, it would be highly interesting to investigate whether the clinical benefits observed with tumor‐treating fields (TTF) therapy for glioblastoma could also result in improved outcomes for GSM. The final analysis of the EF‐14 trial, which demonstrated significant survival benefits for glioblastoma patients treated with TTF, was reported by Stupp et al. Unfortunately, in our study cohort on GSM, only 37 patients were treated after the publication of this landmark paper, and none of these cases included TTF therapy. Consequently, we do not have any cases in our cohort to evaluate the potential benefits of TTF for GSM [43]. Currently, there are no clinical trials specifically recruiting GSM. A number of prospective clinical trials are combining TMZ with targeted therapies such as Iplimumab or Sorafenib in pooled cohorts of glioblastoma and GSM patients; however, no results have been published yet [39, 40]. Table 3 summarizes the current clinical evidence based on larger retrospective cohort studies or cancer database analyses.

**TABLE 3:** Previous clinical studies on gliosarcomas.

Author, country and year	Study type	*N*	Age (median)	Primary GSM	Secondary GSM	Surgery	RT	TMZ‐CTX	OS (months)	PFS (months)	Prognostic factors
Present study	Multi‐institutional cohort study	170	58.0	153 (90.0%)	17 (10.0%)	160 (94.1%)	142 (83.6%)	113 (66.5%)	12.3 median	6.6 median	**MVA:** **OS:** Age, STR, GTR, pRT, TMZ‐CTX, *MGMT* promoter methylation, tumor size **PFS:** GTR, pRT, TMZ‐CTX, tumor size
Amer et al., Housten, TX, USA, 2022	Single‐institutional cohort study	94	59.0	70 (74.5%)	24 (25.5%)	82 (87.2%)	79 (84.0%)	86 (91%)	16.8 median	4.8 median	**UVA:** **OS:** RT
Castelli et al. Rennes, France 2016	Multi‐institutional cohort study	75	60	N/A	N/A	66 (88.0%)	72 (96%)	64.8%	13.0	N/A	**MVA:** **OS:** RT dose > 54 Gy, treatment at recurrence **DFS:** RT dose > 54 Gy, adjuvant chemotherapy
Jin et al., Stanford, CA, USA, 2016	Single‐institutional cohort study	71^a^	61.9	53	15	88.9%	67.4%	60.0%	9.8 (1° GSM) 7.6 (2° GSM)	6.45 (1°GSM) 5.0 (2°GSM)	**MVA:** **OS:** TMZ, GTR **PFS:** TMZ
Adeberg et al., Heidelberg, Germany, 2016	Single‐institutional cohort study	37	62	37	0	34 (92.0%)	37 (100%)	25 (67.6%)	13.4	7.8	**MVA:** **OS:** KPS > 70, TMZ **PFS:** KPS > 70
Frandsen et al., Salt Lake City, UT, USA, 2018	National Cancer Database analysis	1102	61.7 (mean)	N/A	N/A	362 (33%) 665 Unknown (60%)	818 (74%)	N/A	10.7	N/A	**MVA:** **OS:** Age, Sex, GTR, trimodal therapy, insurance status Charlson Comorbidity index
Kozak et al., Madison, WI, USA 2009	SEER database analysis	353	63	N/A	N/A	90.9%	74.5%	N/A	9.0	N/A	**MVA:** **OS:** Age, tumor location, surgery, RT

Abbreviations: DFS = disease‐free survival, GSM = gliosarcoma, GTR = gross total resection, KPS = Karnofsky Performance Status, *MGMT* = O6‐methylguanine‐DNA methyltransferase, MVA = multivariable Cox regression analysis, N/A = not available, OS = overall survival, PFS = progression‐free survival, pRT = postoperative radiotherapy, RT = radiotherapy, STR = subtotal resection, TMZ = temzolomide, TMZ‐CTX = temozolomide‐based chemotherapy, UVA = univariable analysis.

^a^ Included 3 patients not identifiable as primary or secondary gliosarcoma.

### 4.3 Limitations

The present study carries the limitations inherent to retrospective cohort studies. Data on clinical symptoms, tumor location, treatment side effects, and further details on the RT parameters (volumes and dose distributions) were not available. Information on performance status, as one of the most important prognostic factors, was missing in our cohort [41]. Additionally, the cohort of biopsy alone patients was very small. It may clinically be seen as very likely that performing a surgical resection on an infiltrative and aggressive malignant brain tumor measuring 4.6 cm on average should improve survival. Still, the small sample size in the biopsy only group may bias the finding of an improved OS through surgery over biopsy alone. Moreover, the performance status may be the underlying confounder behind the shorter survival in the biopsy only group as patients with poor performance status were not subject to surgical resections (STR, GTR). The missing data could include underlying mediators of patient outcomes and provide rationales for treatment decisions. Furthermore, sampling effects of patients fit for trimodal therapy may bias the results on survival benefits. Interobserver variabilities in neuropathological diagnosis may lead to misclassifications, particularly in older cases.

## 5 Conclusions

GSM represent a rare and distinct subtype of glioblastoma carrying a dismal prognosis. To our knowledge, this is the largest multi‐institutional cohort study investigating outcomes and prognostic factors for GSM. The aggressive trimodal therapy, comprising surgical excision, pRT, and TMZ‐based chemotherapy appears to have the most beneficial effect on survival in GSM patients. Smaller tumor size, younger age, and methylated *MGMT* promoters are associated with improved survival., Future collaborative efforts are warranted to further distinguish GSM as an independent tumor entity and investigate GSM‐directed therapies.

## Author Contributions

**Siyer Roohani:** conceptualization (equal), data curation (lead), investigation (lead), visualization (lead), writing – original draft (lead). **Maximilian Mirwald:** data curation (equal), investigation (equal), writing – review and editing (equal). **Felix Ehret:** formal analysis (equal), visualization (supporting), writing – review and editing (equal). **Christoph Fink:** data curation (equal), writing – review and editing (equal). **Laila König:** data curation (supporting), formal analysis (supporting), writing – review and editing (equal). **Jana Käthe Striefler:** data curation (supporting), formal analysis (supporting), writing – review and editing (equal). **Noelle Samira Jacob:** data curation (equal), formal analysis (supporting), writing – review and editing (equal). **Ilinca Popp:** data curation (supporting), formal analysis (supporting), writing – review and editing (equal). **Johannes Steffel:** data curation (supporting), formal analysis (supporting), writing – review and editing (equal). **Jolina Handtke:** data curation (supporting), formal analysis (supporting), writing – review and editing (equal). **Noa Marie Claßen:** data curation (supporting), formal analysis (supporting), writing – review and editing (equal). **Titus Rotermund:** data curation (supporting), formal analysis (supporting), writing – review and editing (equal). **Daniel Zips:** conceptualization (supporting), formal analysis (supporting), writing – review and editing (equal). **Peter Vajkoczy:** formal analysis (supporting), writing – review and editing (equal). **Ulrich Schüller:** data curation (supporting), formal analysis (supporting), writing – review and editing (equal). **Mateusz Jacek Spałek:** data curation (equal), formal analysis (equal), writing – review and editing (equal). **David Kaul:** conceptualization (lead), formal analysis (equal), project administration (lead), writing – review and editing (equal).

## Ethics Statement

The study was conducted in accordance with the Declaration of Helsinki. The study was approved by the local Institutional Review Board (Ethikkomission der Charité‐Universitätsmedizin Berlin) and permission was granted for the retrospective analysis of patient‐related data in accordance with federal state regulations.

## Conflicts of Interest

The authors declare no conflicts of interest.

## Data Availability

Data available on request from the corresponding author.
